# Nickel Binding to the c-Src SH3 Domain Facilitates Crystallization

**DOI:** 10.2174/0109298665417324250929120040

**Published:** 2025-10-08

**Authors:** Xander Calicdan, Oriana S. Fisher, Byung Hak Ha, Titus J. Boggon, Amy L. Stiegler

**Affiliations:** 1 Department of Molecular, Cellular and Developmental Biology, Yale University, New Haven, CT, USA;; 2 Department of Molecular Biology and Biochemistry, Wesleyan University, Middletown, CT, USA;; 3 Department of Pharmacology, Yale University, New Haven, CT, USA;; 4 Department of Molecular Biophysics and Biochemistry, Yale University, New Haven, CT, USA

**Keywords:** SH3 domain, c-Src tyrosine kinase, nickel coordination, crystal structure, anomalous signal, dimerization

## Abstract

**Introduction:**

Numerous X-ray crystal structures of the c-Src SH3 domain have provided a large sampling of atomic-level information for this important signaling domain. Multiple crystal forms have been reported, with variable crystal lattice contacts and chemical crystallization conditions.

**Materials and Methods:**

We crystallized the c-Src SH3 domain in a crystallization buffer containing NiCl_2_.

**Results:**

A unique crystal structure of the Src SH3 domain in the trigonal space group *H*3_2_ is determined to 1.45 Å resolution. Crystal packing and anomalous scattering reveal that this crystal form is mediated by two ordered nickel ions provided by the crystallization buffer. Nickel coordination occurs in a 2:2 stoichiometry, which dimerizes two SH3 domain monomers across a pseudo-twofold rotation axis and involves the native N-terminal c-Src SH3 amino acid sequence, a surface-exposed histidine residue, and ordered water molecules.

**Discussion:**

This study provides an example of metal-mediated crystallization and metal binding by N-terminal protein residues, contrasting with the Amino-Terminal Copper and Nickel Binding (ATCUN) motif.

**Conclusion:**

Alternative avenues help widen the potential for future crystallography-based studies of the c-Src SH3 domain.

## 1 INTRODUCTION

Cellular signaling proteins are frequently composed of multiple modular domains with independent folds and functions [1]. Among these protein domains, the Src Homology 3 (SH3) domain is one of the most abundant [2]. As its name implies, the SH3 domain was first identified as a conserved region shared among the amino acid sequences of cellular Src (c-Src) tyrosine kinase and the related cellular Abelson tyrosine kinase (c-Abl), phospholipase C, and the adapter protein Crk [3]. In total, 298 SH3 domains were recently reported in 221 human proteins [4]. SH3 domains are also identified in all eukaryotes as well as in some prokaryotes and viruses [4]. These domains typically mediate protein-protein interactions and affect enzyme regulation, substrate binding, protein localization, and motility, regulating pathways that impact numerous cellular processes, including proliferation, migration, endocytosis, and actin cytoskeleton rearrangement [5].

The SH3 domain is a small protein fold comprised of approximately 60 amino acids. The first experimental three-dimensional structures of SH3 domains were from the Src Family Kinases (SFKs) Fyn [6] and c-Src [7], and from alpha-spectrin [8]. Together, these structures revealed a conserved SH3 fold comprising two small beta sheets with loops of variable lengths. This fold typically recognizes polyproline-II helical P-x-x-P motifs (or simply 'PxxP', where x is any amino acid) but can also bind other protein targets in atypical interactions [2, 9-11]. SH3 domain binding to polyproline motifs has been well studied using structural techniques, including X-ray crystallography, which have collectively helped resolve the binding site, specificity determinants, and bidirectionality of peptide binding [7, 12-15].

The founding SH3 domain was identified in c-Src [3] and has been shown to play a major role in autoinhibition of its kinase activity. In addition to the SH3 domain, SFKs include three other conserved domains: Src homology 1 (SH1), which contains the kinase domain; Src homology 2 (SH2), which binds directly to phosphorylated tyrosine residues; and a short Src homology (SH4) region at the N-terminus, which is lipid modified [16]. In the absence of activating signals, intradomain interactions maintain Src in an autoinhibited, or “closed,” conformation. Specifically, the SH2 domain binds an inhibitory phosphotyrosine (pY-527) in the C-terminal tail, while the SH3 domain binds the linker between SH2 and kinase domains at a non-proline helical-like sequence [17]. In this conformation, the SH3 and SH2 domains interact with the kinase domain and restrict it to its inactive conformation. In response to signaling, these SH3- and SH2-mediated autoinhibitory restraints are released, and the kinase domain adopts its active conformation. The SH3 domain of SFKs binds protein targets including Proline-Enriched Phosphatase (PEP) [18], the adapter protein Crk-II [19], the Nef protein of Human Immunodeficiency Virus-1 (HIV-1) [20], the 3BP1 RhoGAP [21], Choline Kinase alpha (ChoKα) [22], and beta-arrestin [23].

The three-dimensional structure of the isolated SH3 domain of c-Src has been reported by numerous X-ray crystallographic and NMR studies [24]. Among the X-ray crystal structures available for Src SH3 in the Protein Data Bank, space group identity, unit cell dimensions, and/or lattice contacts are often replicated, presumably owing to the similarity in crystallization conditions or in the sequence of the recombinant protein. Specifically, several reported crystal forms of the Src SH3 domain require divalent nickel ions for crystallization and are examples of the phenomenon of metal-mediated protein crystallization [25]. The use of metals to facilitate protein crystal lattice formation has long been observed and explored as a potential systematic approach to improve the proclivity of a protein to form a crystalline lattice [26-31]. In this approach, divalent metal ions are coordinated directly by protein atoms, often through histidine-derived nitrogen or cysteine-derived sulfur, and create sites of crystal lattice contacts [29, 31].

Here, we report an X-ray crystal structure of the c-Src SH3 domain in space group *H*3_2_, which has not previously been observed. In the structure, we find lattice contacts mediated by interchain nickel coordination, in another example of metal-mediated crystallization of the Src SH3 domain protein. This metal coordination is achieved through the native protein sequence and demonstrates that native amino acids can serve as nickel-coordinating residues to facilitate crystallization. This crystal packing arrangement may widen the potential for future crystallography-based studies of the c-Src SH3 domain.

## 2 MATERIALS AND METHODS

### 2.1 Expression Construct and Expression

Human c-Src (UNIPROT ID: P12931) cDNA encoding the SH3 domain residues 85-143 was PCR-amplified using Platinum *Taq* HiFi polymerase (ThermoFisher) using a forward oligo containing an NdeI restriction site and encoding for a Tobacco Etch Virus (TEV) protease recognition site, and a reverse oligo containing an XhoI restriction site. The PCR product and the pET-28a(+) plasmid were separately double-digested with NdeI/XhoI in CutSmart buffer (New England Biolabs), and ligated together using T4 DNA ligase (New England Biolabs) to yield an expression construct for N-terminal His_6_-tagged protein with both thrombin and TEV protease recognition sequences. A positive clone was verified through whole-plasmid sequencing (Plasmidsaurus) (Table **S1**). Protein expression was carried out in Rosetta (DE3) *E. coli* cells transformed with the expression plasmid. Cells were cultured in the presence of 25 µg/ml kanamycin and 17 µg/ml chloramphenicol at 37°C, shaking at 200 rpm in Miller's LB Broth (Fisher Scientific) to an OD_600_ of 0.8, then cooled to 18°C prior to induction of protein expression with 0.2 mM Isopropyl β-D-1-thiogalactopyranoside (IPTG). Expression was conducted overnight (approximately 20 hours) at 18°C, shaking at 200 rpm. Cells were harvested by centrifugation.

### 2.2 Protein Purification

Cells were resuspended in lysis buffer (500 mM NaCl and 50 mM HEPES, pH 7.5) and then flash-frozen in a dry ice/ethanol bath. The sample was thawed, and lysozyme was added to a final concentration of 0.1 mg/mL, followed by two more freeze/thaw cycles. The sample was lysed *via* sonication for 3 cycles of 2 minutes using 10-second on/off pulses at 35% with a half-inch probe (Q500, QSonica). DNase I (bovine pancreas, GoldBio) was added at a concentration of 10 Units/ml and incubated for 10 minutes on ice. The total lysate was clarified by centrifugation for 1 hour at 4°C at 32,928 x *g*. Clarified lysate was applied to 1 ml Ni-NTA agarose beads (Qiagen) in a sealed gravity flow column and incubated rocking for 1 hour at 4°C. The flow-through sample was collected, and the beads were washed with wash buffer (lysis buffer containing 20 mM imidazole). Recombinant Tobacco Etch Virus (rTEV) protease was added to the bead-bound His_6_-tagged Src SH3 protein and incubated overnight at 4°C. The liberated untagged c-Src SH3 domain protein was then collected from the bead by washing with a 1 mL aliquot of wash buffer and further purified by size exclusion chromatography (Superdex 75 Increase 10/300 GL) in a buffer containing 150 mM NaCl and 20 mM Tris, pH 7.5. The purified protein was concentrated using a spin concentrator (Amicon Ultra 3 kDa MWCO, Millipore Sigma) to a final concentration of 11 mg/mL. Protein concentration was determined using a NanoDrop (Thermo Fisher), A280 nm blanked with size exclusion buffer using an extinction coefficient of 16,960 M^-1^ cm^-1^ calculated using the primary amino acid sequence of the protein.

### 2.3 Protein Crystallization

Purified c-Src SH3 domain protein at 11 mg/mL was crystallized in hanging drops *via* vapor diffusion at room temperature in 24-well VDX plates (Hampton Research) over 500 µl of reservoir solution. Crystallization drops contained 1 µl of protein with 1 µl of reservoir buffer containing 5 mM Nickel chloride, 1.7 M Ammonium Sulfate, 10% glycerol, and 0.1 M HEPES, pH 7.5. Crystals developed a morphology of rectangular prisms with typical edge dimensions ranging from 25 to 100 µm and appeared after 1 to 3 days. Crystals were harvested, washed in a cryopreservative solution containing reservoir solution and 20% glycerol, and flash-cooled in liquid nitrogen by manual plunging (Table **S2**).

### 2.4 X-ray Data Collection and Processing

X-ray diffraction data were collected at Brookhaven National Laboratory National Synchrotron Light Source II beamline 17-ID-2 (FMX) [32]. Crystal mounting, raster scanning, centering, and data collection were each performed automatically. Data were collected at a wavelength of 0.979315 Å. Autoprocessing was performed in the autoPROC pipeline [33], which utilizes XDS for indexing and integration [34]. Individual datasets from a single crystal comprise 1200 images (4800 images in total) with a 0.01-second exposure and an oscillation range of 0.2° per image for a total rotation range of 240°. Reflection data from 4 individual crystals were input to Aimless for scaling and merging [35]. An anomalous signal was automatically detected *via* XDS in each of the 4 datasets; thus, Friedel pairs were treated separately. Both intensities (*I*(+) and *I*(-)) and structure factors (*F*(+) and *F*(-)) were output to MTZ. A resolution cutoff of 1.45 Å was determined based on a combination of values of *I*/σ*I* > 1.5, CC_½_ > 0.5, and *R*_pim_ < 0.5, using the guidelines of [35]. Crystals belong to space group *H*3_2_ with unit cell dimensions a = 63.77 Å, b = 63.77 Å, c = 271.71 Å, α = β = 90°, γ = 120°. Phenix Xtriage analysis [36] reveals that the diffraction data contain an off-origin native Patterson peak of height 31% due to translational non-crystallographic symmetry (tNCS) (Table **1** and Table **S3**).

### 2.5 Structure Determination, Model Building, and Refinement

The crystal structure was determined by molecular replacement in Phaser [37] using as the search model a previously published crystal structure of the chicken c-Src SH3 domain truncated to residues 86-140 and lacking hydrogen atoms (PDB: 4JZ4 chain A [25]). Translational NCS (tNCS) was detected automatically and applied during Phaser with NMOL equal to 2 and an intermolecular vector of (1, 0, 0.5). A molecular replacement solution containing 4 copies of the SH3 domain was identified with a top TFZ score of 10.9 and an eLLG of 1070, initial *R*_work_ of 30.2%, and *R*_free_ of 32.1%. Autobuilding was performed by Phenix autobuild [38] to 227 residues in 4 chains and 169 waters with *R*_work_ = 25.2% and *R*_free_ = 26.53%. Manual model building was performed in Coot [39] and refinement in Phenix [36] using occupancy refinement and anisotropic *B*-factor refinement for both protein and nickel atoms. Waters were built automatically during autobuild and refinement, refined with isotropic *B*-factors, and then manually inspected and edited in Coot. Glycerol molecules from the cryopreservative solution were modeled manually and refined with isotropic *B*-factors. Nickel atoms were modeled into the anomalous difference density. Secondary structure definitions are obtained using the DSSP method [40]. The free set for *R*_free_ calculation was randomly selected and flagged as 5% of reflections using the Phenix reflection file editor (Table **2**).

Phased anomalous difference maps were calculated in the Phenix utility phenix.find_peaks_holes [36] using the protein model phases combined with the structure factors *F*(+) and *F*(-) from the diffraction data. Two strong anomalous peaks, with heights of 58.9 σ and 43.5 σ, were used to guide the modeling of two nickel atoms. These Ni^2+^ sites were validated using the CheckMyMetal server [41] and the statistics given in Table **3**. Refinement of the anomalous scattering factor *f*” was calculated in Phenix to a value of 1.745 e- (analysis as per [42] and [43]), which is consistent with the theoretical *f*” value of 1.943 e- for Ni at wavelength 0.9793 Å (12,660 eV) obtained *via* X-ray absorption edge tabulations at http://www.bmsc.washington.edu/scatter/AS_index.html [44]. A third anomalous peak of 6.9 σ was present; however, a nickel atom was modeled and refined to an occupancy of less than 0.2 with high *B*-factors and poor coordination. Therefore, this third nickel ion was omitted from the final model. Structures are illustrated with CCP4mg [45] and ChimeraX [46]. All crystallography software was compiled by SBGrid [47].

## 3 RESULTS

### 3.1 Crystallization and Structure Determination

**Figure 1:**
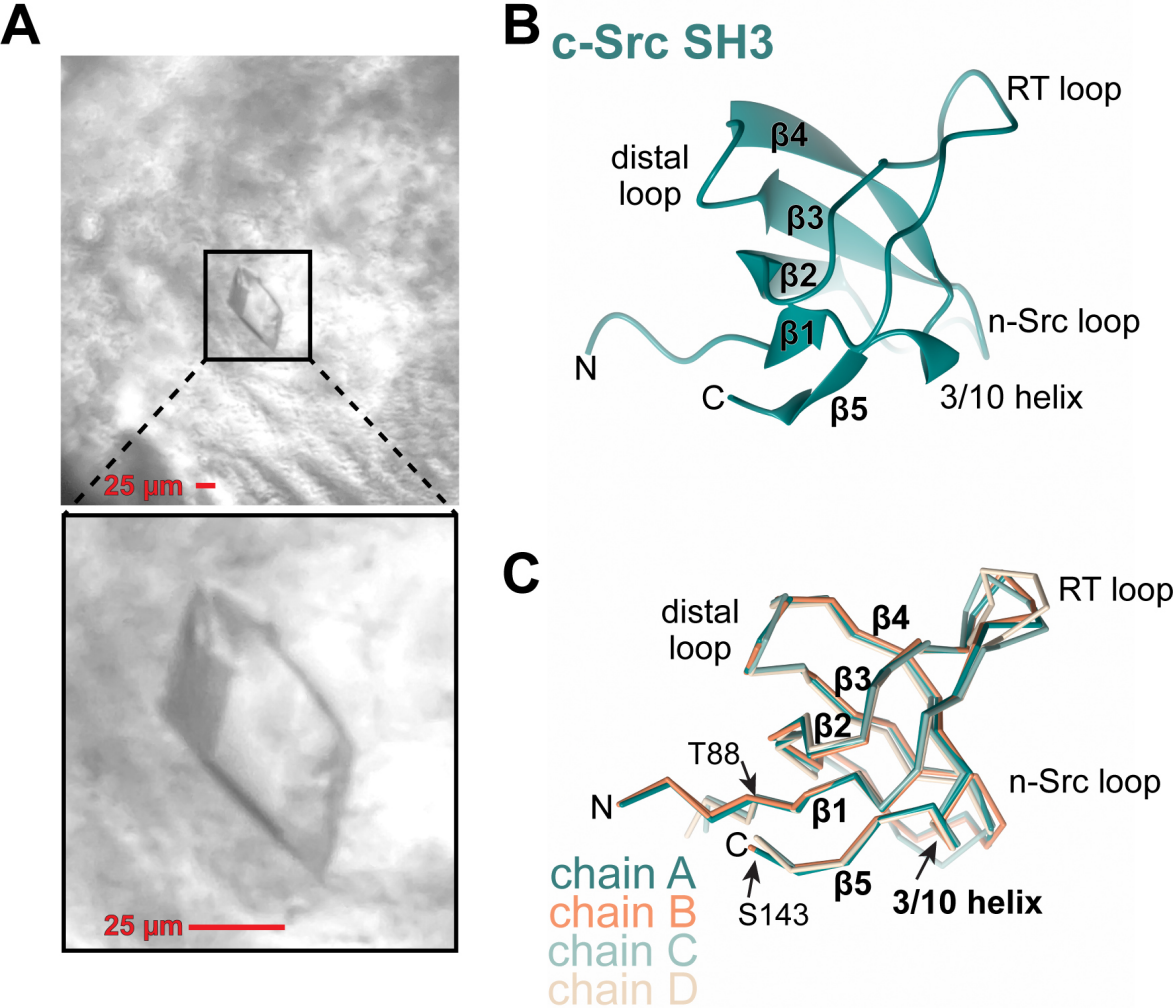
Crystal structure of c-Src SH3 domain in *H*3_2_ crystal form. (**A**) Representative crystals of the c-Src SH3 domain grown for this study. A scale bar representing 25 µm is included in each panel. The average crystal has an edge dimension of 25–100 µm and fits within a 0.05–0.1 mm CryoLoop (Hampton Research). (**B**) Ribbon diagram of the c-Src SH3 domain crystal structure from this study. Shown is chain A. (**C**) Superposition of the structures of each chain of the c-Src SH3 domain from this study. Shown is the Cα trace of each chain, colored dark teal (chain A), orange (chain B), light teal (chain C), and tan (chain D). The positions of Thr-88 and Ser-143 are indicated with arrows. In (**B**) and (**C**), the secondary structure elements, loops, and amino- and carboxy-termini are named and labelled.

We set out to reproduce published crystallization conditions curated in the Protein Data Bank for the isolated c-Src SH3 domain protein for didactic purposes. Among those tested, crystallization conditions containing a cocktail of 1.4 - 1.8 M ammonium sulfate, HEPES pH 7.5, and 5 mM NiCl_2_ in the reservoir solution yielded single crystals with rectangular prism morphology with edge length averaging between 25 - 100 µm despite heavy precipitate also being observed in the drops (Figure **1A**, Table **S2**). We observed that when NiCl_2_ was omitted from crystallization conditions, crystal growth was not achieved. Crystals were harvested from the drop, washed in reservoir solution, cryopreserved, and cryocooled in loops for X-ray data collection at the FMX beamline at NSLS-II. Datasets from four individual crystals were merged to achieve a multiplicity of 52.6. Consequently, the anomalous signal improved and was detected automatically during data processing (Table **1** and Table **S3**).

The structure of the c-Src SH3 domain was determined to 1.45 Å resolution by molecular replacement (Table **2**). Crystals belong to space group *H*3_2_ with unit cell dimensions a = b = 63.77 Å, c = 271.71 Å, α = β = 90°, γ = 120°, and four molecules per asymmetric unit. The *H*3_2_ space group differs from the anticipated space groups *P*2_1_2_1_2_1_ (PDB: 4jz4 and 4omo [25] and 4rtz, unpublished) or *P*2_1_ (PDB: 4rtx, unpublished) of the previously reported c-Src SH3 crystals grown in similar NiCl_2_-containing crystallization conditions. Examination of the Protein Data Bank reveals that no available crystal structure of the Src SH3 domain adopts the *H*3_2_ space group or unit cell dimensions of our crystal form. Furthermore, interface search performed in the 'Protein Interfaces, Surfaces and Assemblies' (PISA) server [48] of crystal lattice contacts in the *H*3_2_ crystal form reveals no common lattice contact in any available crystal structure of c-Src SH3 domain, including the *P*2_1_2_1_2_1_ or *P*2_1_ crystal forms. Thus, this previously reported crystallization condition yielded a unique crystal lattice for the c-Src SH3 domain.

### 3.2 SH3 Domain Structure

We performed model building and refinement of the c-Src SH3 domain structure in space group *H*3_2_. As our structure confirms, the c-Src SH3 domain structure consists of two antiparallel β-sheets formed by strands β1 - β2 - β5 and strands β3 - β4. These strands are connected by the long RT-loop (β1 - β2), n-Src loop (β2 - β3), distal loop (β3 - β4), and a 3_10_ helix (β4 - β5) (Figure **1B** and **1C**) using nomenclature according to previous studies [6]. Chains A, B, and C are fully modeled from residues 85-143, while chain D lacks strong electron density for residues 115-119 (in the n-Src loop), which remain unmodelled. The four copies align closely with one another: upon superposition of residues Thr-88 through Ser-143, the structural alignment yields R.M.S.D. values of 0.38 - 0.99 Å over 54 Cα positions (Figure **1C**) [49]. Small positional changes in the RT and n-Src loops are the most pronounced differences between the modelled chains. Comparison of the four copies in the asymmetric unit also revealed differences in the N-terminal residues, specifically, Gly-85, Val-86, and Thr-87. These variations in conformation are due to nickel binding, which is discussed in detail below, and explain why the asymmetric unit is composed of four individual non-symmetric chains. Our c-Src SH3 structure also closely aligns with previously determined structures, with R.M.S.D. values ranging from 0.4 to 1.5 Å over 54 - 59 Cα positions for many of the available Src SH3 domain structures in the PDB (Figure **S1**, Dali server [50]). We therefore consider that the unique lattice contacts in our *H*3_2_ crystal form are not attributed to large conformational changes or structural rearrangements in the Src SH3 domain protein.

### 3.3 Nickel Coordination in the Asymmetric Unit

**Figure 2:**
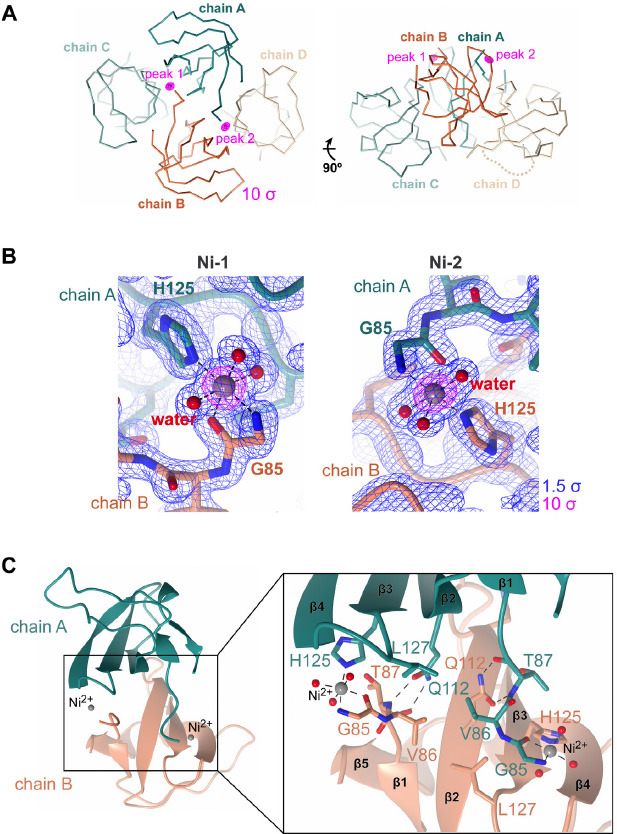
Nickel coordination *via* the c-Src SH3 domain. (**A**) The asymmetric unit containing four chains of c-Src SH3 domain (Cα traces) and phased anomalous difference map contoured to 10 σ (pink) shows two strong peaks (peak 1 and peak 2) arranged between chains A (dark teal) and B (orange). The two views are related by a 90° rotation about the x-axis away from the reader. (**B**) Final refined 2*F*_o_-*F*_c_ map (blue, contoured to 1.5 σ) and phased anomalous difference map (pink, contoured to 10 σ). Ni^2+^ atoms are drawn as grey spheres, and water molecules are red spheres. (**C**) Details of nickel binding. Left panel: overview of the chain A/ chain B interface. Zoom-in: details of the interface, including intrachain nickel coordination (black dashed lines) by Gly-85 and His-125.

Since a strong anomalous signal was detected in the X-ray diffraction data (Table **1** and Table **S3**), we computed phased anomalous difference maps, which revealed two large anomalous peaks with heights of 58.9 σ and 43.5 σ (Figure **2A** and Table **3**) at locations where auto-building had initially modeled water molecules. However, after refinement of these waters, the positive *F*_o_-*F*_c_ difference map remained highly elevated (not shown), suggestive of the presence of a non-water atom (such as a metal) at these positions. As 5 mM NiCl_2_ was required for crystal growth (Table **S2**) and nickel has a sufficiently high theoretical anomalous *f*” atomic absorption value of 1.951 [44] electrons at the X-ray collection wavelength (0.98 Å), we modeled and refined nickel atoms at these anomalous peak positions (Figure **2B**). The two nickel sites are located at the interface of Src SH3 chains A and B in a 2:2 stoichiometry. The nickel atoms bridge the A and B chains by coordination of the nitrogen and carboxyl oxygen atoms of the N-terminal residue Gly-85 of one chain and the imidazole epsilon nitrogen atom of His-125 on β3 in the neighboring chain. The crystallization pH value of 7.5 is favorable for metal coordination by histidine, since its side chain, with a pKa value of 6.0, will be at least partially deprotonated. Octahedral metal coordination is completed by three water molecules (Figure **2B**). The refined nickel-ligand bond distances (1.97 - 1.99 Å) are within the expected range (Table **3** and Figure **S2A** and **S2B**). Both nickel sites are validated by CheckMyMetal Binding Site Validation [41] and FindGeo [51] servers (Table **3**).

We further examined the 2:2 stoichiometry interaction between the Ni^2+^ ions and chains A and B of the SH3 domain. These chains are related by non-crystallographic twofold rotational symmetry, and the interface buries a surface area of approximately 440 Å^2^ per chain. In addition to the intrachain Ni^2+^ coordination, the interface features hydrogen bonding between Gln-112 and Thr-87, as well as van der Waals interactions including Val-86 and Leu-127 near the nickel binding site (Figure **2C**). There is also a small patch of van der Waals interactions involving Asn-115 and Thr-117 on the opposite side of the chains A/B interface (Figure **S3**). The nickel-mediated symmetrization aids in lattice formation by stabilizing the interactions between chains A and B within the asymmetric unit.

### 3.4 Role of Nickel in Lattice Formation in the *H*3_2_ Space Group

**Figure 3:**
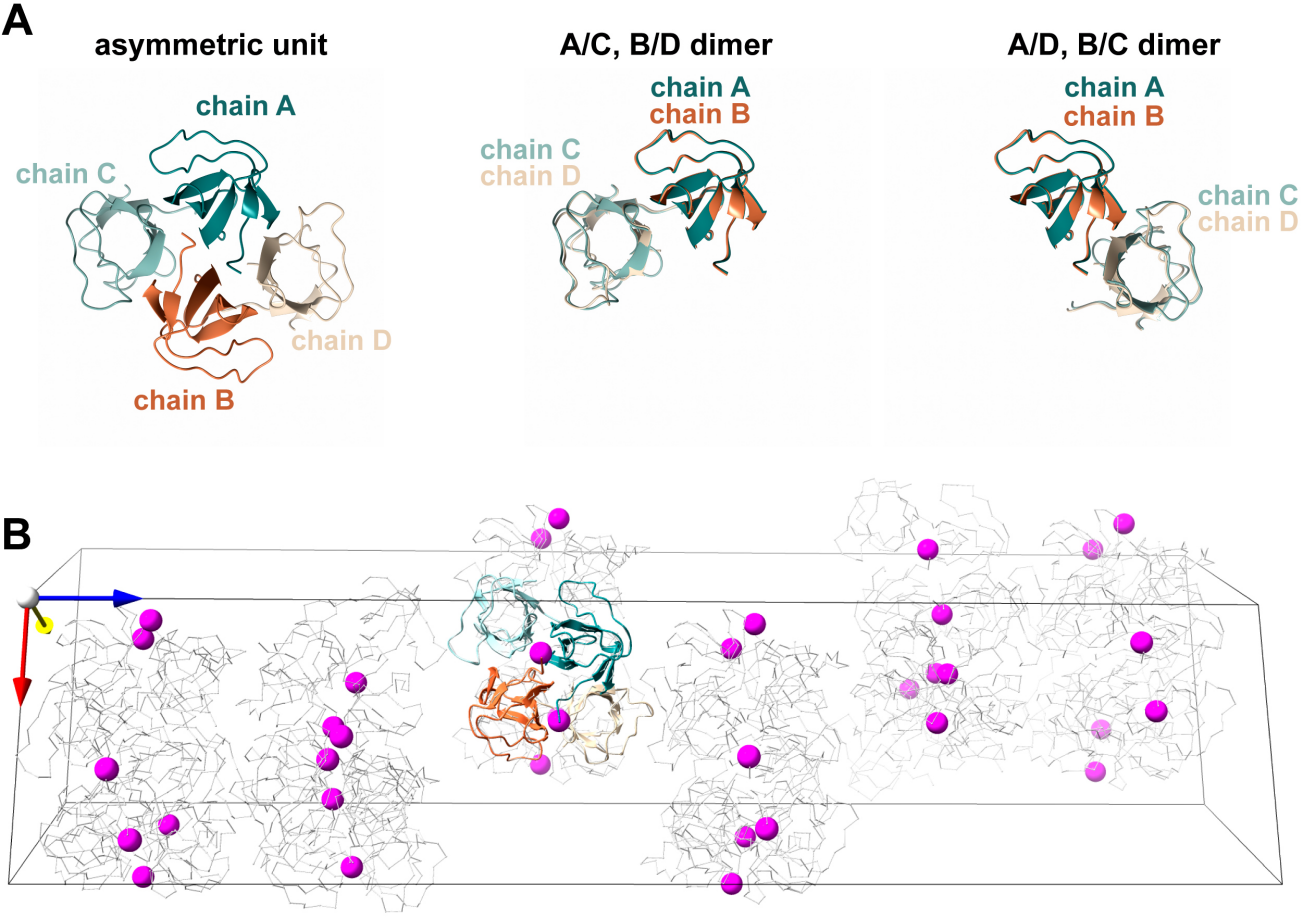
Lattice contacts in the c-Src SH3 *H*3_2_ crystal form. (**A**) Left panel: asymmetric unit arrangement of four copies of the c-Src SH3 domain. Two types of dimers are depicted in the center and right subpanels. (**B**) Expanded view of the unit cell, which is drawn as a box. The nickel sites are indicated as pink spheres. One copy of the asymmetric unit is drawn with protein ribbons and colored as in part (A). The other protein monomers are drawn as gray Cα traces. The orientation axes x (red), y (yellow), and z (blue) are indicated. The nickel sites bridge monomers A and B in the asymmetric unit, but do not further aid in crystal packing between asymmetric units.

We then more closely examined the other crystal contacts in this *H*3_2_ c-Src SH3 crystal form. The four molecules in the asymmetric unit form two types of equivalent dimers: one between chains A/C and B/D, which buries approximately 500 Å^2^ per monomer, and a second between chains A/D and B/C, which buries an average of 380 Å^2^ per monomer (Figure **3A**). Each of these dimer sets is related by non-crystallographic two-fold rotational symmetry. An interface search of the available structures in the Protein Data Bank for each of these dimers using the PISA server [48] reveals no equivalent Src SH3 interface. Thus, the lattice formation in the *H*3_2_ space group is unique among available crystal structures of the Src SH3 domain. The binding of Ni^2+^ across the interface of chains A and B bridges these dimer pairs to help stabilize the *H*3_2_ crystal lattice. Next, examination of the global crystal packing within the unit cell reveals that Ni coordination aids in asymmetric unit formation but is not involved in the packing of the asymmetric units into the unit cell (Figure **3B**). Taken together, these observations support the conclusion that nickel aids in the formation of the asymmetric unit and represents an example of metal-mediated protein crystallization whereby crystal lattice contacts are formed through nickel coordination.

To further study this phenomenon, we attempted to detect nickel-induced dimer formation of the SH3 domain by comparing the elution profile from size exclusion chromatography and migration through semi-native SDS-PAGE [52, 53] in the absence and presence of a five-fold molar excess of Ni^2+^ from NiCl_2_; however, neither method yielded conclusive results. Therefore, whether nickel-induced dimerization occurs *in vitro* prior to crystallization or *in situ* in the crystallization drop during vapor diffusion remains unresolved.

### 3.5 Comparison with the Amino-terminal Copper and Nickel (ATCUN) Binding Motif

The *H*3_2_ crystal form of the c-Src SH3 domain differs from those previously reported, despite being grown under similar crystallization conditions containing NiCl_2_. To understand this discrepancy, we examined the protein constructs used for crystallization. We designed the expression plasmid to encode a TEV protease recognition sequence (Glu-Asn-Leu-Tyr-Phe-Gln-Gly) occurring C-terminal to the His_6_-tag, which is absent from the parental pET-28 vector. After TEV proteolysis of the purified protein, which occurs between the Gln-Gly peptide bond of the recognition sequence, the resulting N-terminus bears the sequence Gly-Val, which is equivalent to Gly-85 and Val-86 in the native Src sequence (Table **S1**). This cloning scheme yields an all-native amino acid sequence following TEV proteolysis and facilitates the Ni^2+^ ion-mediated crystal form (Figures **2B** and **2C**).

**Figure 4:**
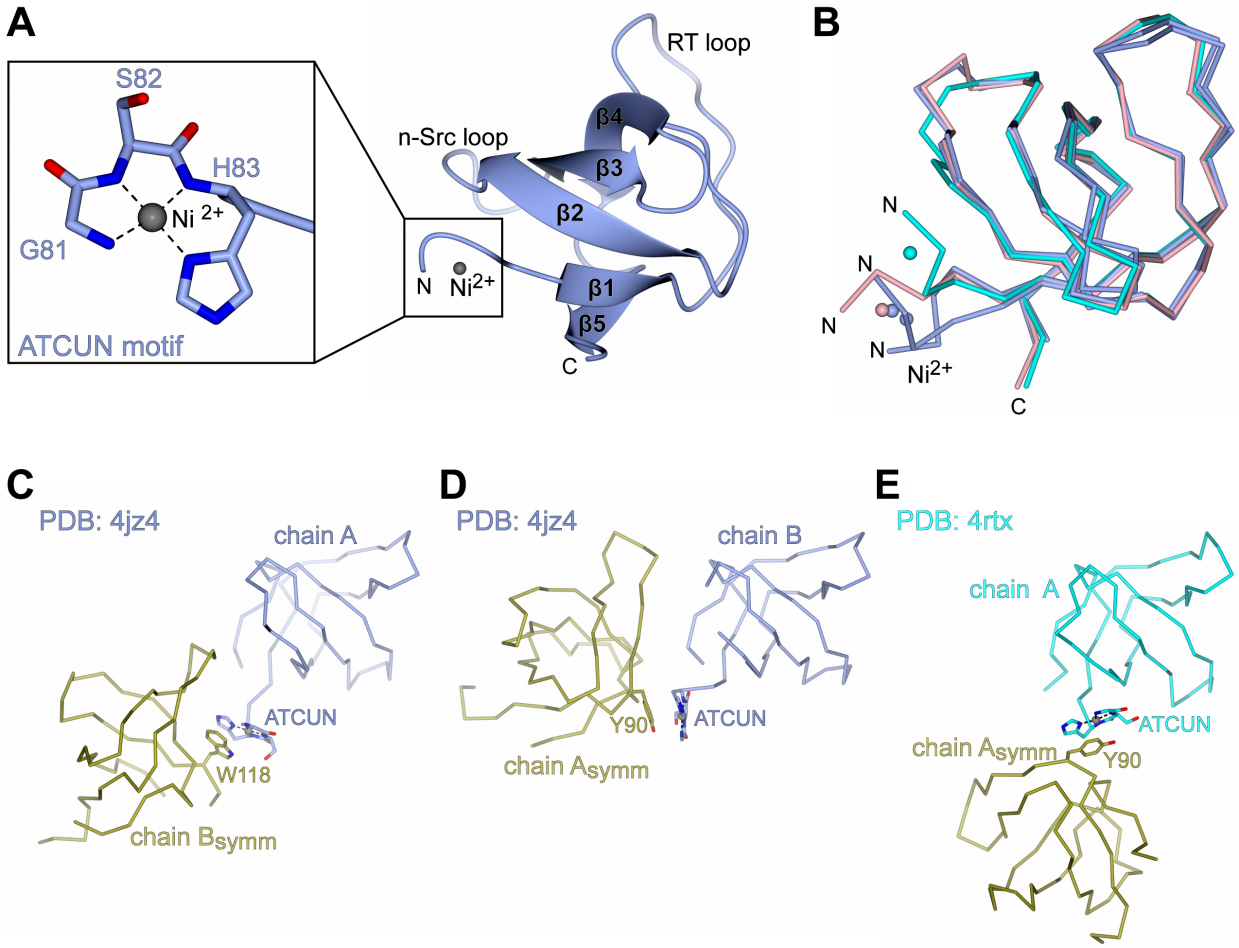
Nickel coordination by the c-Src SH3 domains containing an ATCUN motif. (**A**) The ATCUN motif Gly-Ser-His at the N-terminus of the Src SH3 domain protein (PDB: 4jz4, [25]) coordinates nickel in square planar geometry. (**B**) Superposition of c-Src SH3 domains containing ATCUN and coordinating nickel. Included are PDBs 4jz4 (light blue, chains A and B, [25]), 4rtz (light pink, *P*2_1_2_1_2_1_, unpublished), and 4rtx (cyan, *P*2_1_, unpublished). PDB 4omo [25] is equivalent to 4jz4 and is not shown. The nickel sites are depicted as spheres and colored according to the corresponding PDB entry. (**C**) Symmetry-related π-stacking interaction involving the nickel-binding ATCUN motif of chain A (light blue) and the sidechain of Tyr-90 in symmetry-related chain B (chain B_symm_, gold). PDB: 4jz4 [25]. (**D**) Symmetry-related π-stacking interaction involving the nickel-binding ATCUN motif of chain B (light blue) and the sidechain of Trp-118 in a symmetry-related chain A (chain A_symm_, gold). PDB: 4jz4 [25]. (**E**) Symmetry-related π-stacking interaction involving the nickel-binding ATCUN motif of one chain (cyan) and the sidechain of Tyr-90 in a second chain (gold). PDB: 4rtx (unpublished). For comparison, the orientation of the main SH3 domain molecule is equivalent in parts C, D, and E.

We next compared the Ni^2+^ binding by the c-Src SH3 in the *H*3_2_ space group with the Ni^2+^ binding in the previously reported crystal structures of Src SH3 from similar NiCl_2_-containing crystallization conditions and at similar pH values of 7.5 (PDB: 4jz4 and 4omo [25], 4rtz (unpublished), and 4rtx (unpublished)). In these structures, Ni^2+^ ions are modelled and refined, and each chain of c-Src SH3 coordinates a single Ni^2+^ ion *via* the N-terminal residues Gly-Ser-His, which form a so-called 'Amino Terminal Copper and Nickel' (ATCUN) binding motif [54]. The classic monomeric ATCUN motif is comprised of the amino-terminal tripeptide consensus sequence (H_2_N)-x-x-His (where x is any amino acid and histidine is at position 3), which is found in the naturally occurring ATCUN site in proteins, including human and bovine serum albumin, where it is used for copper transport in the blood [55]. The ATCUN motif is also used as an engineered short peptide motif well-suited for metal coordination [56], which can chelate divalent metals at very high affinities (100 fM) [57]. In the bona fide ATCUN-motif containing Src SH3 protein structures, the nickel ion is coordinated by the ATCUN motif of a single chain in distorted square planar geometry *via* four-nitrogen chelation from the backbone nitrogen atoms of Gly-81, Ser-82 and His-83 and an imidazole nitrogen from the sidechain of His-83 with bond distances ranging from 1.83 - 2.02 Å (Figure **4A** and Figures **S2C** and **S2D**). This Gly-Ser-His motif is present at the N-terminus of these c-Src SH3 domain proteins as a result of cloning artifacts and thrombin protease cleavage, and for c-Src SH3, it is a non-native amino acid sequence. This differs from our interchain nickel coordination mediated by native amino acid sequences at the N-terminus Gly-85 and Val-86 and the sidechain of His-125 across the dimer interface (Figures **2** and **S2A** and **S2B**). Thus, nickel coordination by Src SH3 contrasts binding of metals by the ATCUN motif-containing proteins.

Despite coordinating nickel in a distinct manner from what is observed here, the ATCUN motifs in the previous nickel-containing Src SH3 domain crystal structures still contributed to crystal packing. Among these ATCUN structures are different N-terminal conformations with flexibility in the positioning of the Ni-bound ATCUN motifs with respect to the folded portion of the SH3 domain (Figure **4B**). In addition, these Src SH3 crystals adopt different space groups (*P*2_1_2_1_2_1_ and *P*2_1_), and in the case of *P*2_1_2_1_2_1_, two distinct unit cells with different packing interfaces are observed. It has been postulated that this N-terminal flexibility contributes to the different crystal packing among these earlier structures [58]. In contrast to the *H*3_2_ crystal form, the previous Ni-bound ATCUN motifs do not bridge monomers by direct nickel coordination; instead, the ATCUN motif promotes crystal packing by forming π-stacking interactions with one of two aromatic residues in neighboring symmetry-related chains, either Trp-118 (Figure **4C**) or Phe-90 (Figures **4D** and **4E**). The fourth ATCUN-containing SH3 structure, PDB 4rtz (unpublished), is a co-crystal structure with a PxxP peptide. In this crystal form, the peptide contributes to crystal packing, and the ATCUN-Ni participates in minor van der Waals interactions with a neighboring chain yet does not undergo π-stacking (not shown). Thus, flexibility in the N-terminal ATCUN motifs promotes different crystal packing. The *H*3_2_ crystal form represents a further exploration of Src SH3 N-terminal-mediated nickel coordination.

### 3.6 The Src SH3 *H*3_2_ Crystal Form is Not Compatible with Binding Extended PxxP Peptides

**Figure 5:**
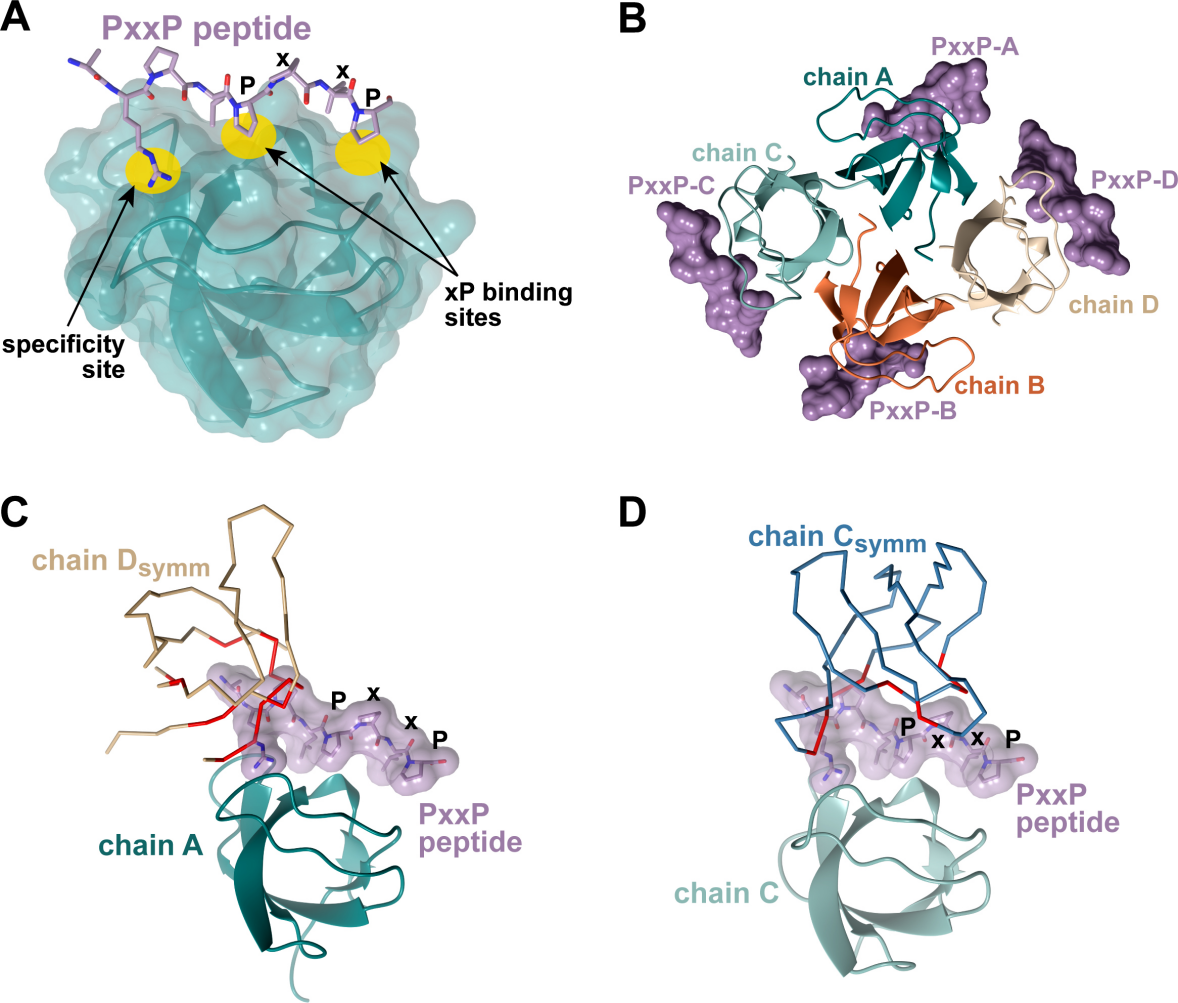
Modeling PxxP binding onto c-Src SH3 domains in *H*3_2_ space group. (**A**) Co-crystal structure of Src SH3 domain (teal, drawn in ribbon and semi-transparent surface) bound to the VSL12 peptide (in purple) (PDB ID: 4rtz, unpublished). Peptide binding sites on SH3 are highlighted in yellow and labeled. (**B**) Asymmetric unit of c-Src SH3 *H*3_2_ crystal structure. The PxxP peptide (purple surface) is modeled onto each chain by superposition of the complex from (A) with each individual chain of the asymmetric unit, with R.M.S.D. values ranging from 0.7 to 1.0 over 57 equivalent Cα positions. (**C**) and (**D**) Modeling reveals that the predicted PxxP binding sites are blocked by symmetry-related molecules in the *H*3_2_ crystal form. Residues in the symmetry mates (Chain D_symm_ (C) and Chain C_symm_ (D)) with predicted steric overlaps with the modeled PxxP peptide are colored red on the Cα trace (calculated in MolProbity [72]). (C) c-Src SH3 chain A (teal ribbon) and its symmetry-related chain D (D_symm_, tan Cα trace). Chain B shares a similar interface with a symmetry-related chain C (Figure **S4A**). (**D**) c-Src SH3 chain C (light cyan ribbon) and symmetry-related chain C (C_symm_, grey-blue). Chain D shares a similar interface with a symmetry-related chain D (Figure **S4B**).

We next assessed whether the c-Src SH3 *H*3_2_ crystal lattice is compatible with typical PxxP peptide binding, to ask if this crystal form might be amenable to in-crystal binding studies [59]. SH3 domains bind typical PxxP-containing peptides *via* a binding pocket between the RT- and n-Src loops. This binding site can be broken up into three sub-pockets: two shallow Proline-binding (xP) pockets that accommodate the minimum PxxP binding motif and a third specificity site that binds residues in an extended peptide (Figure **5A**) [10]. We superposed a co-crystal structure of the Src SH3 domain bound to the VSL12 peptide (PDB ID: 1rtz, unpublished, R.M.S.D. 1.1 - 1.4 Å over 56 equivalent Cα positions) onto each chain of our crystal structure and examined the positions of the modelled PxxP peptide. Within the asymmetric unit, PxxP peptide binding to each of the four chains is compatible with the SH3 domain arrangement (Figure **5B**). Next, we extended the asymmetric unit by generating symmetry mates using crystal symmetry and examined the symmetry-related lattice contacts. We observe two distinct lattice contact arrangements between chains in the asymmetric unit and symmetry-related molecules. First, chain A interacts with symmetry-related chain D (Figure **5C**), and this lattice contact arrangement is shared between chain B and symmetry-related chain C (Figure **S4A**). When modeling a PxxP peptide bound to the SH3 domain, we observe that its lattice contact site sterically clashes with the predicted position of the PxxP peptide. Second, chain C interacts with a symmetry-related chain C (Figure **5D**), which is shared between chain D and symmetry-related chain D (Figure **S4B**), but distinct from that observed for chains A and B (Figure **5C**). In this arrangement, PxxP peptide binding to the typical binding site is also prevented by lattice contacts. We thus conclude that the standard PxxP peptide binding to the SH3 domain at the xP and specificity sites is incompatible with the formation of the *H*3_2_ crystal lattice since these sites in the SH3 domain are involved in crystal lattice contacts.

## 4 DISCUSSION

In this study, we describe a new crystal form of the c-Src SH3 domain protein that is dependent on interchain metal-mediated coordination and unique crystal contacts, thereby expanding the crystallization chemical space and lattice types for this important regulatory domain. This is achieved due to the native N-terminus of the isolated c-Src SH3 protein creating a previously unobserved binding site for Ni^2+^ ions. This finding offers potential benefits in biotechnology. For example, proteins in the environment of a crystal lattice are used as targets in drug discovery, like crystal fragment-based screens [60]. The ability to obtain diverse lattice types for different protein targets affords a wider scope of available binding sites for fragment-based screening [61].

In this example, metal binding facilitates protein crystallization by providing direct lattice contact sites. Multiple systematic approaches to improve the propensity of purified proteins to form well-ordered crystals for X-ray diffraction studies, also called “crystal lattice engineering,” have been proposed. For example, protein modification tools, including mutational surface entropy reduction [62] or enzymatic lysine methylation [63], have been described as approaches to promote protein crystal lattice formation. Other approaches, sometimes termed synthetic (or mutational) symmetrization, involve engineered surface amino acid mutations to promote protein self-multimerization in a symmetric arrangement. This includes mutations to drive disulfide bond formation [64, 65], surface leucine zippers [66], and metal coordination [29, 67].

Here, we provide evidence of metal-mediated symmetrization in the absence of the additional step of creating synthetic (or mutational) sites. For the c-Src SH3 domain, this metal-mediated crystallization instead uses native amino acid residues: an N-terminal glycine residue that is the result of engineering of the protein expression construct, and a surface-exposed histidine in a second molecule. This raises the possibility of metal-mediated symmetrization in otherwise recalcitrant protein samples, as has been observed previously (for example, [26, 68]). Thus, inclusion of metal ions during initial crystallization screening may help proteins self-associate into a crystal lattice. Common divalent metal salts include NiCl_2_, CdCl_2_, CoCl_2_, and CuSO_4_, which are readily accessible in many protein crystallography labs. Additionally, the absorption edge wavelengths of these common metal ions are readily achievable at standard synchrotron beamlines, which may allow for an anomalous signal to be recorded and used for metal ion placement in the three-dimensional model as well as phasing for macromolecular structure solution [43, 69].

## STUDY LIMITATIONS

However, despite our observation here for Src SH3 domain protein, there may be limitations to the general use of nickel and other metal ions to facilitate novel crystal lattice formation for proteins that may not have accessible metal-binding residues on their surface. High throughput crystal screening efforts with multiple divalent cations, and/or extensive surface mutagenesis efforts, may be required in order to identify a metal-coordinated crystal form.

Lastly, our study demonstrates that continued research on the SH3 domain can yield new insights that expand the current knowledge of this important modular protein domain. Currently, a search of the term “SH3 domain” in the PDB yields over 6,000 entries for X-ray crystal structures, highlighting the abundance of structural information for this ubiquitous domain. SH3 domain structures were once referred to as the “darlings of the structural biology set” [24] due to their prominence. Importantly, the function and activity of SH3 domains were first elucidated, in part, by structural biology studies that yielded three-dimensional models of these domains. For example, three-dimensional structures have helped uncover the site of PxxP ligand binding, specificity determinants, autoinhibition within the Src family kinases, and atypical binding with non-PxxP binding partners to alternative sites on the SH3 domain [9, 17]. Such sustained structural studies of SH3 domains have proven beneficial. The three-dimensional structure of a Src family kinase SH3 domain was first observed in 1992-1993 [6, 7], and the Src SH3 domain continues to be a current target of structural studies [70, 71].

## CONCLUSION

Previous X-ray crystal structures of the c-Src SH3 domain have helped define this important signaling domain by providing molecular-level details of its domain fold, binding properties, and specificity-determining features. Here, we present a unique crystal form of the c-Src SH3 domain that is mediated by nickel ion coordination by the native Src protein sequence. This expands the crystallization space for the Src SH3 domain and provides an example of metal-mediated protein crystal lattice formation.

## Figures and Tables

**Table 1:** X-ray diffraction data collection and processing statistics.

Wavelength (Å)	0.979315
Number of crystals merged	4
Resolution range (Å)	31.95 - 1.45 (1.48 - 1.45)
Anomalous resolution limit (Å)^+^	2.00
Space group	*H*3_2_
Cell dimensions a, b, c (Å) α, β, γ (°)	63.77, 63.77, 271.71 90, 90, 120
Number of reflections	-
Total observations	2,030,203
Unique (anomalous)	73,036
Unique (non-anomalous)	38,628
Completeness (%)	100 (100)
Anomalous completeness (%)	100 (100)
Redundancy	52.6 (46.8)
Anomalous redundancy	27.2 (23.8)
Mean *I*/σ(*I*)	22.9 (1.8)
Anomalous Mean d*I*/σ(d*I)*	1.3 (0.89)
Mosaicity (°) of merged data	0.07
CC_1/2_	1.00 (0.784)
*R*_pim_	0.012 (0.509)
Wilson *B*-factor (Å^2^)	23.07

**Note:** Values given in parentheses are for the highest resolution shell.

**Table 2:** Structure refinement.

Resolution range (Å)	31.89 - 1.45 (1.47 - 1.45)
Completeness (%)	99.9 (100)
No. of reflections, working set	73,036 (2,734)
No. of reflections, test set	3,450 (137)
Reflections, test set size (%)	4.72 (5.0)
Final *R*_work_	16.25 (27.88)
Final *R*_free_	19.61 (37.45)
No. of non-H atoms	-
Protein	1,888
Metals (Ni^2+^)	2
Waters, other solvent	163, 54
Total	2,107
R.M.S. deviations from ideality*	-
Bonds (Å)	0.005
Angles (°)	0.879
Average *B*-factors (Å^2^)	-
Protein	30.70
Metals (Ni^2+^)	24.62
Waters, other solvent	38.87, 58.29
Overall	32.07
MolProbity Statistics	-
Ramachandran plot	-
Favored regions (%)	98.19
Allowed regions (%)	1.81
Outliers (%)	0.00
Clashscore	2.63
Unmodelled residues (%)	2.12% (5 out of 236 total residues)
PDB Code	9OFX

**Note:** Values given in parentheses are for the highest resolution shell.

*Phenix Geometry restraints library.

**Table 3:** Nickel site information.

Anomalous scatterer	Nickel	
Photon energy (eV)	12,660	
Theoretical *f*” (e-)	1.943	
Refined *f*” (e-)	1.745	
	-	
Nickel sites	Ni-1	Ni-2
Peak height (σ)*	58.9	43.5
CheckMyMetal^+^ statistics	-	
*B*-factor (Å^2^)	22.0	27.2
Valence	2.7	2.8
Geometry	Octahedral	Octahedral
R.M.S.D. Geometry^+,#^		
Angles (°)	4.3	6.0
Bonds (Å)	0.178	0.250
Metal-ligand distances (Å)		
Gly-85 N	1.97	1.97
Gly-85 O	1.97	1.98
His-125 N (Nε2)	1.99	1.99
Water	1.97 (1)	1.97 (4)
Water	1.98 (2)	1.97 (5)
Water	1.99 (3)	1.97 (6)

**Note:** *Anomalous peak heights (σ) calculated in Phenix (phenix.find_ peaks_holes)

^+^ CheckMyMetal Server [41]

^#^ FindGeo Server [51]

## Data Availability

X-ray crystal structure model coordinates have been deposited in the RSCB Protein Data Bank (PDB) under accession code 9OFX (https://doi.org/10.2210/pdb9OFX/pdb). X-ray diffraction images are available online at SBGrid Data Bank under dataset number 1158 (10.15785/SBGRID/1158). The expression plasmid map and full plasmid sequence are provided as supplementary materials.

## LIST OF ABBREVIATIONS

SH3: Src Homology 3
ATCUN: Amino-Terminal Copper and Nickel Binding
PxxP: Polyproline-II helical P-x-x-P

## SUPPLEMENTARY MATERIAL

1. Supplementary Tables, Figures, and Figure Legends file (Calicdan_Stiegler_PPL_submission-supplemen-tary.docx). 2. PDB submission validation report file (Calicdan-Stiegler-PDB-9OFX-Validation-Report.pdf). 3. Expression plasmid map file (Calicdan-Stiegler-hSrc-SH3NH_85-143_pET28a_Map.tiff). 4. Expression plasmid full sequence file (Calicdan-Stiegler-hSrc-SH3NH_85-143_pET28a.txt). Supplementary material is available on the publisher’s website along with the published article.
