# Supplementary material for: Nickel Binding to the c-Src SH3 Domain Facilitates Crystallization
Source: Protein Pept Lett. 2025 Oct 8;32(9):679–92. doi: 10.2174/0109298665417324250929120040 (PMC12582252; doi:10.2174/0109298665417324250929120040)
Supplement: Supplementary file 1 [file PPL-32-9-679_SD1.pdf]

Supplementary Materials

Nickel Binding to the c-Src SH3 Domain Facilitates Crystallization

Xander Calicdan[1], Oriana S. Fisher[2], Byung Hak Ha[3], Titus J. Boggon[3,4,*] and Amy L. Stiegler[3,*]

[1]*Department of Molecular, Cellular and Developmental Biology, Yale University, New Haven, CT, USA;* [2]*Department of Molecular Biology and Biochemistry, Wesleyan University, Middletown, CT, USA;* [3]*Department of Pharmacology, Yale University, New Haven, CT, USA;* [4]*Department of Molecular Biophysics and Biochemistry, Yale University, New Haven, CT, USA*

Table S1. Macromolecule production information.

Source organism	*Homo sapiens*
DNA source	cDNA
Expression vector	pET-28a(+)
Plasmid construction method	PCR and restriction-ligation
Forward primer<br>(NheI and TEV site)	5' - GGATTCCATATGGAGAACCTGTACTTTCAA<br>GGCGTGACCACCTTTGTGGCCC - 3'
Reverse primer<br>(STOP codon and XhoI)	5' - CCGCTCGAGCTAGGAGGGCGCCACATAGTTG - 3'
Expression host	*E. coli* Rosetta(DE3)
Expression details	Induced with 0.2 mM IPTG at OD_{600} = 0.8, 18°C, 20 hr
Complete amino acid sequence of the protein produced	MGSSHHHHHHSSGLVPRGSHMENLYFQGVTTFVALYDY<br>ESRTETDLSFKKGERLQIVNNTEGDWWLAHSLSTGQT<br>GYIPSNYVAPS
Complete amino acid sequence of the protein after TEV proteolysis	85 - GVTTF VALYDYESRT ETDLSFKKGE RLQIVNNTEG<br>DWWLAHSLST GQTGYIPSNY VAPS - 143

Table S2. Crystallization information.

Method	Hanging drop vapor diffusion
Plate type	VDX™ Plate with sealant (Hampton Research)
Temperature (°C)	25º C (room temperature)
Protein concentration	1.8 mM, 11.9 mg/mL
Buffer composition of protein solution	150 mM NaCl, 20 mM Tris pH 7.5
Composition of reservoir solution	1.7 M Ammonium Sulfate, 5 mM $NiCl_2$, 10% glycerol, 0.1 M HEPES pH 7.5
Volume and ratio of drop	2 µL drop, 1:1 v/v protein to reservoir ratio
Volume of reservoir	500 µl in VDX plate (Hampton Research)
Composition of the cryopreservative	1.7 M Ammonium Sulfate, 5 mM $NiCl_2$, 20% glycerol, 0.1 M HEPES pH 7.5
Drop setting	Manual
Seeding	No

0929-8665/25 **© 2025 Bentham Science Publishers**

Table S3. Additional Data collection and processing statistics.

Diffraction source	BNL NSLS-II 17-ID-2 FMX			
Detector	EIGER 16M			
Numver of crystals merged	4			
Individual crystals	1	2	3	4
Temperature (K)	100	100	100	100
Crystal to detector distance (mm)	200	200	200	200
Total rotation range (°)	240	240	240	240
Rotation per image (°)	0.2	0.2	0.2	0.2
Exposure time per image (s)	0.01	0.01	0.01	0.01
Space group	$H3_2$	$H3_2$	$H3_2$	$H3_2$
Unit cell dimensions<br>a (Å)<br>b (Å)<br>c (Å)<br>α, β, γ (°)	63.760 63.760<br>271.669<br>90, 90, 120	63.976 63.976<br>272.214<br>90, 90, 120	63.976 63.976<br>272.816<br>90, 90, 120	63.867 63.867<br>272.628<br>90, 90, 120
Mosaicity (°)	0.09	0.06	0.07	0.06
Anomalous resolution limit (Å)[+]	2.63	2.40	2.16	2.21

[+]Anomalous signal detected in Aimless (Evans and Murshudov, 2013) where $CC_{anom} < 0.15$

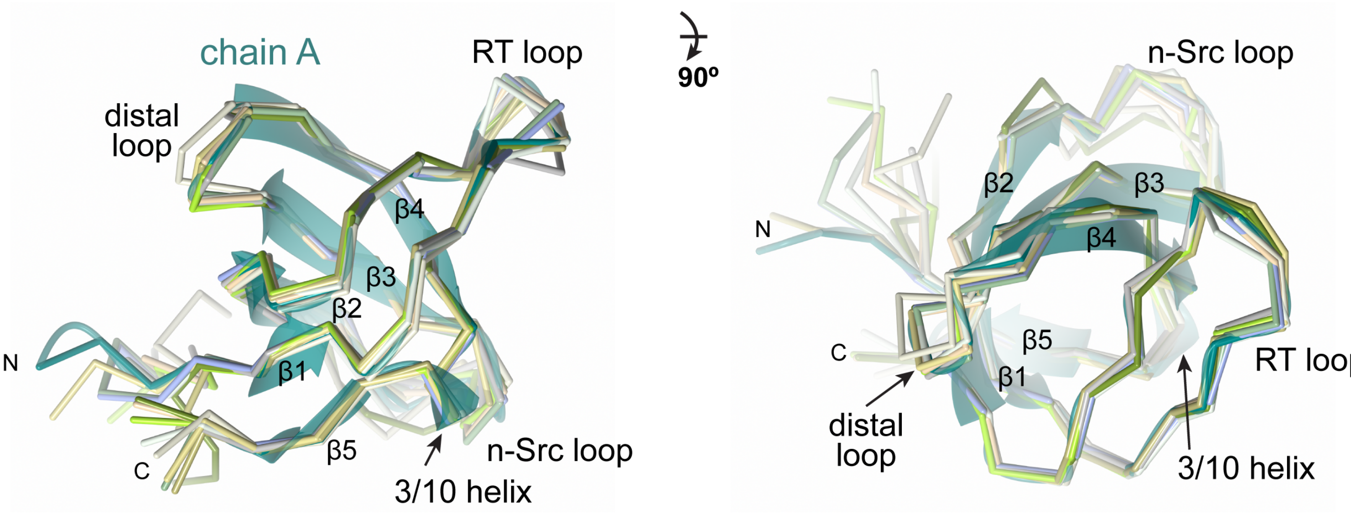

Figure S1. Superposition of 10 representative c-Src SH3 domain structures. Chain A from the crystal structure in this study shown in dark teal ribbons. Superposed are a representative set of c-Src SH3 domain crystal structures from the PDB. Secondary structure elements and loops are labelled. The view in the right panel is related to the left view by a 90° rotation toward the reader about the x-axis. The R.M.S.D. of superposition ranges from 0.55 - 1.09 Å over 58 equivalent Cα positions for the ten representative structures shown. Superpositions were performed using the DALI server (Holm and Rosenstrom, 2010). The ten representative structures are as follows: PDB: 4jz4 (Bacarizo et al., 2014), 4omo (Bacarizo et al., 2014), 4rtx (unpublished), 4rtz (unpublished), 6c4s (Kall et al., 2019), 4hxj (Xiao et al., 2013), 4hvw (Bacarizo and Camara-Artigas, 2013), 5eca (unpublished), 7a3c (unpublished), 7a32 (unpublished).

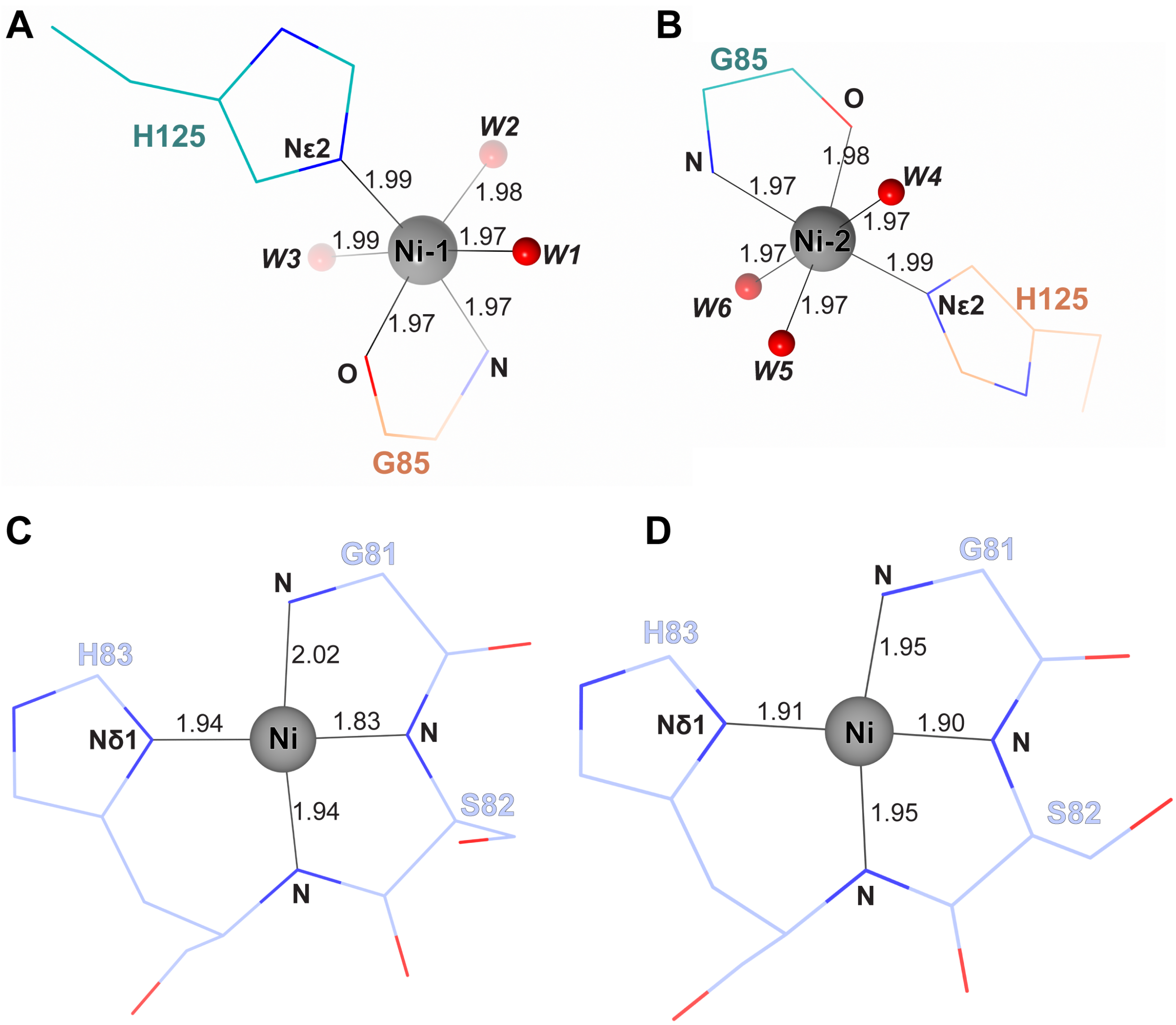

Figure S2. Metal-ligand bond distances. (A) c-Src SH3 structure in $H3_2$ crystal form with octahedral coordination of nickel (Ni-1) by His-125 of chain A (teal), Gly-85 of chain B (orange), and three water molecules (red spheres), showing the metal-ligand distance for each bond. (B) Similar to part (A), the octahedral coordination of nickel (Ni-2) by Gly-85 of chain A (teal), His-125 of chain B (orange), and three water molecules (red spheres). In both panels, the water molecules are numbered according to the final PDB model. These nickel-ligand distances are also listed in Table 5. (C) and (D) Metal-ligand bond distances in classic ATCUN motif bound to nickel in square planar geometry in the Src SH3 structure (PDB: 4jz4 (Bacarizo et al., 2014)) chains A (panel C) and B (panel D). All bond distances are reported by CheckMyMetal server (Gucwa et al., 2023).

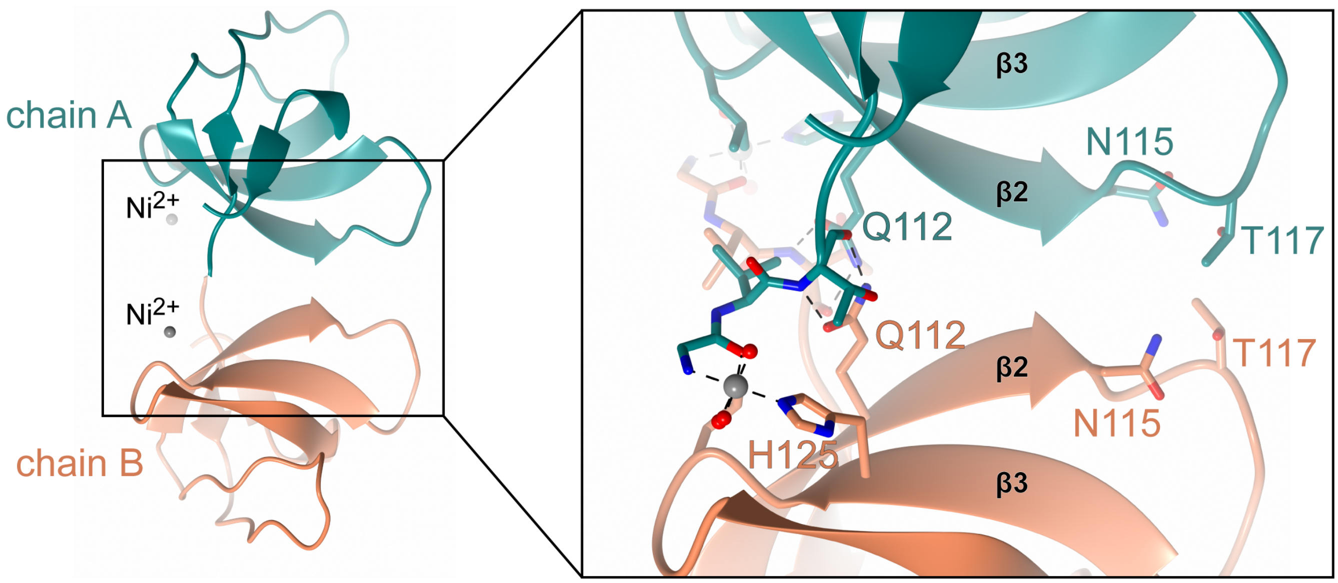

Figure S3. Additional details of the interface between chains A and B in the asymmetric unit. Left panel: Chains A (dark teal) and B (orange) as ribbons and two Ni^{2+} ions (grey spheres) in the asymmetric unit. The view is related to Figure 2c by a 90° rotation to the left on the y-axis. Right panel: Zoom-in view shows the details of the interface including the sidechains of Asn-115 and Thr-117 in both chains which participate in van der Waals interactions between the chains.

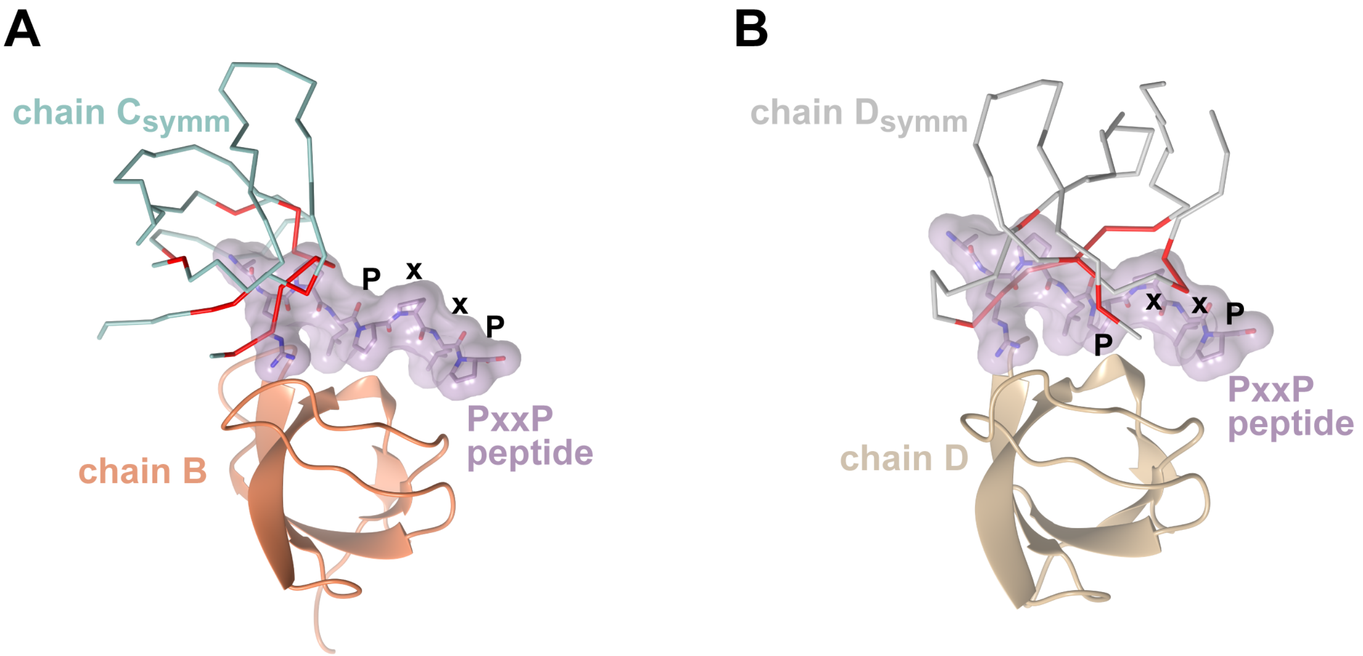

Figure S4. Predicted PxxP peptide binding is blocked by symmetry-related molecules. The predicted PxxP binding sites are blocked by symmetry-related molecules in the *H*3$_2$ crystal form. Related to Figure 5. (A) c-Src SH3 chain B (orange ribbon) and its neighboring lattice partner chain D_{symm} (light cyan Cα trace). This interface is similar to the one shown in Figure 5C. (B) Chain D (tan ribbon) and its neighboring lattice partner chain D_{symm} (grey Cα trace). This interface is similar to the one shown in Figure 5D. In (A) and (B), residues in the symmetry mates (chain D_{symm} (A) and Chain D_{symm} (B)) with predicted serious steric overlaps with the modeled PxxP peptide are colored red on the Cα traces (calculated in MolProbity (Chen et al., 2010)).
